# Biomechanical Aspects of the Foot Arch, Body Balance and Body Weight Composition of Boys Training Football

**DOI:** 10.3390/ijerph18095017

**Published:** 2021-05-10

**Authors:** Joanna M. Bukowska, Małgorzata Jekiełek, Dariusz Kruczkowski, Tadeusz Ambroży, Jarosław Jaszczur-Nowicki

**Affiliations:** 1 Department of Tourism, Recreation and Ecology, University of Warmia and Mazury, 10-719 Olsztyn, Poland; joanna.bukowska@uwm.edu.pl; 2 Department of Ergonomics and Physiological Effort, Institute of Physiotherapy, Jagiellonian University Collegium Medicum, 31-126 Krakow, Poland; malgorzata.jekielek@gmail.com; 3 Faculty of Health Sciences, Elbląg University of the Humanities and Economics, 82-300 Elbląg, Poland; dyrektor@olimpijczyk.gda.pl; 4 Institute of Sports Sciences, University of Physical Education, 31-571 Krakow, Poland; tadek@ambrozy.pl

**Keywords:** foot, ground pressure, body composition, body balance, football players

## Abstract

*Background:* The aim of the study is to assess the body balance and podological parameters and body composition of young footballers in the context of the control of football training. *Methods:* The study examined the distribution of the pressure of the part of the foot on the ground, the arch of the foot, and the analysis of the body composition of the boys. The pressure center for both feet and the whole body was also examined. The study involved 90 youth footballers from Olsztyn and Barczewo in three age groups: 8–10 years, 11–13 years old, and 14–16 years. The study used the Inbody 270 body composition analyzer and the EPSR1, a mat that measures the pressure distribution of the feet on the ground. *Results:* The results showed statistically significant differences in almost every case for each area of the foot between the groups of the examined boys. The most significant differences were observed for the metatarsal area and the left heel. In the case of stabilization of the whole body, statistically significant differences were noted between all study groups. In the case of the body composition parameters, in the examined boys, a coherent direction of changes was noticed for most of them. The relationships and correlations between the examined parameters were also investigated. The significance level in the study was set at *p* < 0.05. *Conclusions:* Under the training rigor, a statistically significant increase in stability was observed with age. The total length of the longitudinal arch of both feet of the examined boys showed a tendency to flatten in direct proportion to the age of the examined boys. Mean values of the body composition parameters reflect changes with the ontogenetic development, basic somatic parameters (body height and weight) and training experience, and thus with the intensity and volume of training. This indicates a correct training process that does not interfere with the proper development of the body in terms of tissue and biochemical composition.

## 1. Introduction

The attempt to achieve the championship forced the coaches to pay more attention to training children and adolescents. This procedure seems to be fundamental, but in practice it very often causes many errors, deformations or even degenerations [1,2]. Most often, in the case of talented youth, there is a quick entry into sport for adults, often with temporary successes of young players. However, they are unprepared physically, mentally, technically and tactically. It is often accompanied by the exhaustion of a young athlete, both physically (injuries, permanent damage to the musculoskeletal system, problems in the field of motor coordination, lack of progress in the field of physical preparation) and mentally [3]. Coordination abilities can be a diagnostic tool for monitoring the dynamics of their development and on the basis of them, conclusions can be drawn about the dynamics of physical health [4]. The current knowledge and many years of training experience, not only in football, clearly show that properly selected methods and forms of training are the key to success. Perfectly matched training loads at individual stages of a player’s development may bring in the future the result of an optimally prepared footballer for a world-class sport fight [3]. Football is a team game in which the players should represent a sufficiently high level of speed, strength and coordination motor skills. The level of these abilities may depend on the task performed on the pitch as well as on the sport advancement [5,6]. Coordination is one of the factors indicating a significant improvement in physical performance. This is confirmed by the directly proportional relationship between muscle strength and neuromuscular coordination [7,8]. The aim of general coordination training is to develop, improve, stabilize and restore coordination skills or performance requirements in order to be able to successfully cope with all motor tasks in sport and everyday life [9]. One of the coordination skills is balance, which is the ability to concentrate on one’s own body [10]. Balance has a direct and significant influence on the ability to dribble [11]. A better balance of the body allows for better results in sports [12]. As a result of the literature review, it can be stated that people from various sports disciplines training at a higher level have better balance than people who are just starting their training [13,14,15]. The cause of problems and at the same time a greater risk of lower limb injuries is overweight. One of the effects of excessive fat mass in the torso reduces the degree of mobility, balance control and a decrease in postural stability [16]. The foot is an important part of the musculoskeletal system. Its function is to support the conditioning of human movement. The foot is influenced by a number of factors that have a positive effect on it or contribute to the formation of defects [17]. The use of the foot as a basic element in practicing football causes it to carry out more work than during everyday activities. Biomechanical loads that the foot is subjected to while kicking a ball, the use of special and specific footwear and the varied terrain of various sports fields (compacted earth, grass, etc.) activate a number of muscles and joints that do not function with the same intensity and mobility in everyday life [18]. Soccer players are exposed to injuries due to overload, discomfort and decreased performance due to shoe design and repetitive plantar loads [19]. The specificity of the sport discipline causes players to have different morphological profiles [17]. Laterality is also a factor that can affect foot function. Khudik, Chikurov, Voynich et al. believe that asymmetry manifests itself in the human body. Cultural factors and genetics influence the asymmetry of a given individual, and Guilherme J. et al. in their research show that training influences the functional asymmetry of the lower limbs in young football players. [20]. During the research, the following hypothesis and purpose of the research was formulated: sports activity related to football training allows for the biological development of a human being in accordance with the norms in the field of body posture and its composition. The aim of the study is to assess the physical aspects of the musculoskeletal system, related to the structure of the foot and the ability to maintain body balance, as well as to analyze the body composition of young football players in the context of football training control.

## 2. Materials and Methods 

### 2.1. Participants

The study included 90 youth footballers from Olsztyn and Barczewo in three age groups: 8–10 years (mean age 9 ± 0.86 years, body weight 33.66 ± 8.51 kg, body height 136.03 ± 10.31 cm), 11–13 years (mean age 12.55 ± 0.63 years, body weight 47.83 ± 7.66 kg, height 159.79 ± 6.72 cm) and 14–16 years (mean age 14.30 ± 0.46 years, body weight 60.08 ± 10.31 kg, height 171.61 ± 6.57 cm). All boys aged 8–16, training in clubs in Olsztyn and Barczewo, who were entered into the games at the province or central level in their age categories, participated in the research. Boys under examination took part in training three times a week. Each training session consisted of a warm-up, improving football skills, shaping motor skills with or without the ball, improving individual and team behavior in specific parts of the game, playing ball, and stretching at the end of training. The research was conducted during the competition period. All respondents are players of the same league games in different age categories. The construction of the training unit between the teams was very similar and adapted to their age. All players declared their right upper and lower limbs as dominant and had no visible dysfunctions in the musculoskeletal system. Parents and trainers gave their written consent to the study. All examined boys were players of the same class of games, at different levels of classification depending on age. All coaches of the studied players have the same game goals: victory in individual matches and, as a result, obtaining the best possible place in the league. The research was conducted on the basis of the consent of the Scientific Research Ethics Committee of the University of Warmia and Mazury in Olsztyn (Decision No. 9/2018). 

### 2.2. Instruments

The body composition analyzer Inbody 270 (Inbody Co. Ltd, Soul, Korea) was used for the research. It is a specialized medical device that uses the bioelectric impedance method to measure body composition using a quantitative method. This method is based on the ability to electrically conduct muscle tissue. Body height was measured with a Soehnle (Soehnle, Gaildorfer Straße 6, 71522 Backnang, Germany) electronic ultrasonic height measuring device. (The height measuring device performs the ultrasound measurement and the built-in tilt sensor helps to measure it precisely. The device transmits data to the computer program Lookin’Body 120 (Included in the package with the Inbody 270 device). For the measurement of foot pressure distribution and balance, the EPSR1 mat (Letsens Group, Letsens S.R.L. Via Buozzi, CastelMaggiore; Bologna, Italy) was used. The 700 × 500 × 5 mm mat is equipped with 2304 pressure sensors located on the active surface. It is a diagnostic device used to evaluate the feet in static and dynamic conditions. The mat is equipped with sensors that collect the measurements for 20 seconds and transfers them to the computer using the Biomech Studio program (Biomech Studio 2.0 Manual, (Letsens Group, Letsens S.R.L. Via Buozzi, CastelMaggiore; Bologna, Italy). The following stabilometric parameters can be measured with the mat:

- COP LF—area of left foot imbalances,
- COP RF—right foot imbalances area,
- body COP—the surface of the body’s center of gravity.

### 2.3. Procedure

**Figure 1:**
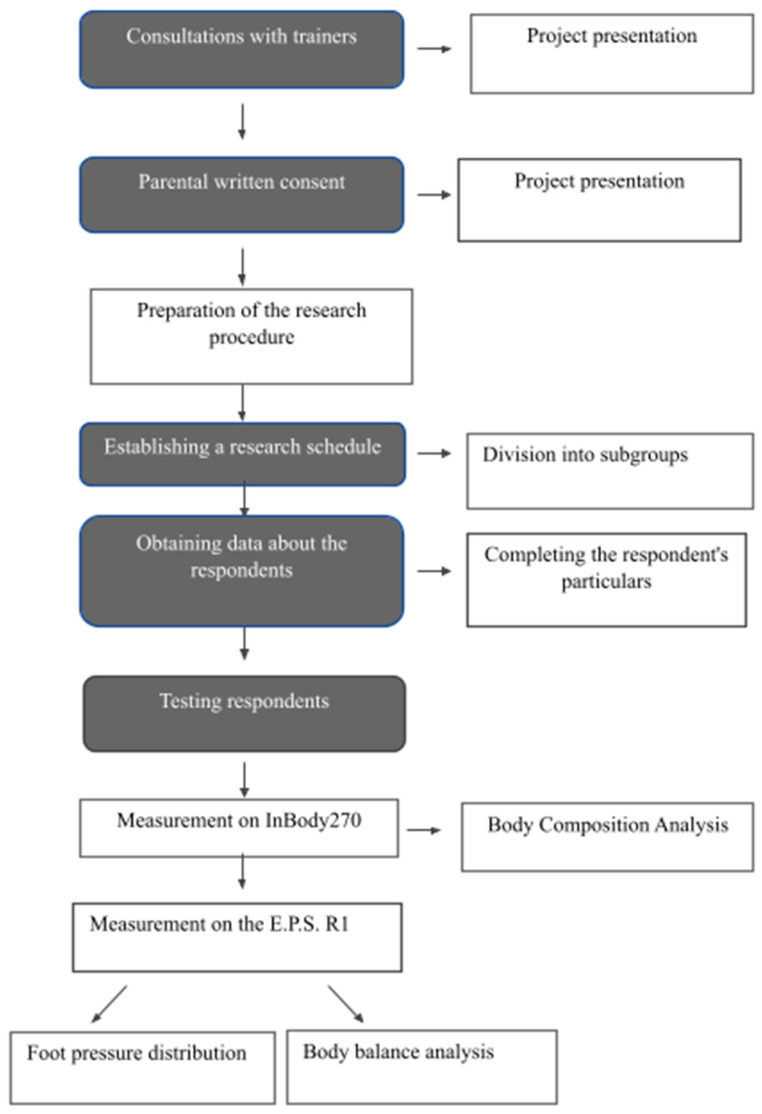
Scheme of the test procedure.

The research was conducted on 5 March 2020, 11 March 2020 and 30 July 2020. The test procedure consisted of several steps. In the initial stages, consultations with trainers and parents were conducted regarding the planned study. The study schedule was also drawn up and parental consent was obtained for the study of boys. Before starting the study, a qualified person entered the data of the test person into the program, such as ID, date of birth and height. The height of the body was checked by the researcher with the help of a measuring rod, keeping an upright posture. Then, after undressing to underwear, removing jewelry and glasses, the erect participant climbed the analyzer platform so that the feet covered as much of the electrodes as possible. The device automatically started measuring the body weight. After completing the measurement, the examined person took the handles of the device in their hands with their thumb touching the upper electrode and the other fingers of the lower electrode. The subject was asked to remove the extended arms from the body so as not to touch the torso, as this could affect the reliability of the results. The feet and hands adhered to the electrodes throughout the examination, and special attention was paid to it. During the composition analysis, the boys’ standards were checked by precisely referring to the body parameters generated by the program for each of them. In order to avoid errors, the tests were carried out in accordance with the procedure enclosed by the manufacturer in the device manual. The examined boys were either fasting or at least 2–3 h after a meal, and also after defecation. In order to optimize the obtained results, the test was performed in the morning, before exercise, approximately 2–3 min after changing from sitting to standing. Earlier bathing was a contraindication to the study, as it accelerates blood flow in the body, which the respondents were aware of. After the end of the test, the data were automatically sent to the Lookin’Body 120 program and the subject put down the handles of the device and left it. Then, the examined boy went to the podographic mat so that his feet were on both sides of the vertical line drawn on the mat. They were then asked to take a few steps to place their feet freely on the mat. The boy stood upright with his arms against his body, staring straight ahead. The signal remained stationary for 20 seconds. At that time, the measurements were made and transferred to a computer system using the Biomech Studio software (Biomech Studio 2.0 Manual). The diagram of the test procedure is shown in Figure 1.

### 2.4. Statistical Analysis

The Shapiro–Wilk test was used to analyze the studied groups of boys categorized according to their age, which indicated non-compliance with the normal distribution for all measurement parameters. Therefore, in further analysis, a statistical nonparametric function was used, which is the rank test of Kruskal–Wallis. In the case of podological and stabilographic measurements, the two-tailed test with Bonferroni’s correction was used due to the mean differences between the groups of boys under study. In the whole work for the characteristics of descriptive statistics, the measures of mean value, median and dispersion of quartile measurement values were used. During the statistical analysis, the Chi square test and the Spearman correlation test were also used to analyze the dependence and correlation between the obtained results. The significance level in the study was set at *p <* 0.05. Statistical analyses were performed using the Statistica program (StatSoft Polska, Kraków, Poland version 13.3).

## 3. Results

During the analysis, differences in the size of the foot arches were noticed for each of the studied groups of young footballers. The mean values of the pressure of the metatarsal area in the group of boys aged 8–10 years indicated significant hollowing of both feet. For the left foot, the value of the pressure area on the metatarsus was only 0.1%, and in the right foot, 0.8%. Taking into account the mean values for the group of boys aged 11–13 years, the left foot was significantly hollow (0.4%), while the right foot was in the range of the average hollow (9.8%). The results for the oldest group of boys aged 14–16 were the highest for the left foot, placing the value in the range of mean arching (12.9%) and the right foot in the range of significant arching (2.3%). The detailed results of the podiatry examination in the studied age groups are presented in Table 1.

Analyzing the results of the stabilographic examination, significant differences were noticed in the size of all parameters defining the field of changes in the position of the entire body, as well as the area for the left and right feet. Taking into account the total parameter of the pressure center of the whole body, the group of the youngest footballers aged 8–10 (360.62 mm^2^) showed the least stability. In the following groups of boys, an increase in the stabilization of body posture was noticed, where in the group of boys aged 11–13 years, the size of the area for the whole body was 197.84 mm^2^, while for the oldest group, aged 14–16, the mean value was 37.76 mm^2^. The results of the stabilographic examination are presented in Table 2.

The obtained results were statistically evaluated. In the podiatry study, statistically significant differences were noticed between the groups of boys, categorized in specific age groups, in almost every case for each area of the foot. The most significant differences were observed for the metatarsal area and the heel in the left foot. In the metatarsal area, significant differences were noted between all the studied groups of young footballers. Referring to the results of the evaluation of the statistical differences in the stabilographic study, significant differences were noted for all parameters. In the case of the summary parameter constituting the stabilization of the whole body, statistically significant differences were noted between all groups of the studied footballers. The results of the analysis of the statistical significance of differences in individual parameters and age groups are presented in Table 3, Table 4 and Table 5. 

In the statistical analysis, differences between the groups of boys were noticed in the size of their body composition parameters. For most of them, there was a homogeneous direction of changes. A total of 80–95% of the examined boys, divided into groups, had measurement values within the normal range (TBW, proteins, FFM, WHR). In the case of the parameters related to total body water (TBW), lean body mass (FFM), skeletal muscle mass (SMM), body mass index (BMI), but also in the case of protein and mineral content in the body, the mean measurement values increased directly proportional to the age of the respondents. The differences in the mean values of the above-mentioned parameters for each group of boys were statistically significant. In the case of the percentage of adipose tissue (PBF) and the degree of obesity (obesity degree), the mean values for the subsequent study groups were inversely proportional. In their case, statistically significant differences were also noted between the boys’ groups. By comparing the mean values of the parameters: total body fat mass (BFM) and the waist–hip index (WHR) and visceral fat (VFL) related to the analysis of the abdominal area, no statistically significant differences were found and the mean values for each group were almost equal. Taking into account the specificity of physical effort, football players require a high ratio of muscle mass to body fat mass, where excess fat mass leads to a significant reduction in exercise capacity. The general body mass index BMI for boys of the youngest age group remained at the lower limit of the normal range (BMI = 17.5), while in the other two groups it was at the lower limits of the normal range: BMI = 18.30 and BMI = 20.20. The gradual increase in the value of the index is closely related to the mass of skeletal muscles, where successively in the age groups of the studied footballers there is an average higher skeletal muscle mass in the body, respectively: SMM: 12.65; 20.80; 28.40. This relationship translates into mean values of lean body mass (FFM). Additionally, in the case of this parameter, the mean value increases in the subsequent age groups, respectively: FFM: 24.75; 38.40; 51.30. The analysis of body composition in terms of water content (TBW), proteins and minerals also follow the principle of a directly proportional increase in mean values in relation to the age of the studied group of boys. The mean values of the percentage of adipose tissue in the subsequent age groups of the boys under study are inversely proportional to the analyzed indicators, respectively: PBF: 22.15; 16.50; 11.30. The characteristics of body composition parameters (InBody) presented in Table 6. 

During the statistical analysis, the relationships between the examined parameters were also examined. Both the relationships between the examined features and the correlation between them were analyzed. For this purpose, the Chi^2^ test and the Spearman correlation test were used. In the group of the youngest boys, a statistically significant relationship and correlation between the body COP and the WHR parameter was observed. The examined boys with an increase in this parameter had a greater problem with balance while standing on both feet. However, a similar correlation was not obtained for the correlation and dependence with BMI, BFM, PBF, VFL and obesity degree. In the age group between 11 and 13 years of age, there was no correlation between body composition related to adipose tissue and body balance. In the group of the oldest boys, a statistically significant relationship and correlation between body COP and BFM was demonstrated. However, it was a negative correlation. The same relationship was noted between body COP and PBF, VFL, WHR and obesity degree. Taking into account the entire sample, there was a statistically significant correlation between body COP and BMI. With the increase in BMI, greater disturbances were noted in the examined boys. The same relationships were noted between body COP and PBF and obesity degree.

## 4. Discussion

Human biological development usually proceeds according to the norms. This enables the comparison of percentiles and the decision of whether the changes comply with the physiological norm or not. When assessing body composition, all parameters in the study group are consistent with the physiological norm. This indicates the correct selection of the examined adolescents and the proper training process, which does not disturb the proper development of the organism in terms of tissue and biochemical composition. The problem of body composition in young footballers was studied by Santos-Silva et al. The 16-week futsal training program contributed to the improvement of body composition and cardiovascular capacity in a group of boys before puberty (7–10 years). There was a significant increase in total body weight (4%), height (3%), lean body mass (8%) and a significant 6% decrease in body fat percentage [21]. The research presented in this study also shows that in older boys (14–16 years old) there was a greater percentage of boys who were below the norm than in the other groups. Similar results were obtained by Ørntoft et al. [22] found that Danish children aged 10–12 engaged in club football (FC) and other ball games (OBGs) had more muscle mass and a lower body fat percentage than children who did not play sports in their free time (NSC). Children participating in club ball games had a higher (*p <* 0.05) lean body weight than NSC and OBG: participation in soccer classes also affects the percentage of body fat. Significant scientific reports indicate the improvement of the body’s ability to maintain balance until the age of 10–12. The differences in the limits of the ability maximization are determined by the measurement method, or rather the conditions for showing the body’s ability to balance. Different limits are indicated by the authors for measurements under static conditions, others under dynamic conditions. In our own study, it was observed that the body balance was better with the increasing duration of the training rigor. The oldest group of the boys under study showed the lowest balance disturbances during the stabilometric test. The research conducted by Lebiedowska M. and Syczewska M. [23] showed that despite changes in the body dimensions of children between 7 and 18 years of age in balance tests, the invariability of the swing amplitude is noticeable, which confirms the view that the same patterns of muscle activation are used in children and adolescents. Different results, but adequate to the authors of this study, were obtained by Riach C.L. and Starkes J.L. [24] who in studies of children (4–13 years old) and adults showed an age-related change in the velocity of the center of gravity and in the position of the feet. The problem of the influence of regular football training on balance was investigated by Olchowiak G. and Czwalik A. [25]. The authors carried out research on women training in football (*n* = 25) and a control group (*n* = 50). In the tests used, statistically significant differences between the groups were obtained. Women training in football showed better postural stability and balance. The study showed that regular training can improve the balance system. The authors’ conclusions are consistent with the results obtained by the authors of this study, as they showed statistically significant differences between the groups in terms of body posture stability. Kumala M.S. et al. [26] also dealt with balance in athletes, comparing the balance between normal and flat feet. None of the tests performed showed statistically significant differences between the groups (*p* > 0.05) in the balance of the body. Jaszczur-Nowicki J. et al. [27,28,29] in their works analyze changes in the balance and distribution of pressure forces on the plantar side of the foot under the influence of various factors. In children, under the influence of an external load (backpack), the results of body balance were statistically significant. They concerned measurements of the area of the center of gravity of the body, the area of the center of gravity of the left foot and the parameter comparing the distance to area ratio. In all these parameters tested, *p <* 0.05 was obtained. The authors obtained statistically significant results in all parameters of the body balance by analyzing the influence of exercise (Harvard Step Test) on the examined parameters in students. The authors of the above studies also analyzed the distribution of pressure forces on the plantar side of the foot; in children, the results indicated that after putting on the backpack for the entire study group, statistically significant differences (*p <* 0,05) were found in the distribution of the foot pressure on the ground in the left foot, forefoot, and heel area. However, in the right foot, this difference was noted for the forefoot and the metatarsus. The *p*-value in these parameters was also below 0.05. On the other hand, among students, when comparing the mean results of measurements at rest and after exercises for the forefoot, the value of the rest vs. the post-training values for the left foot were comparable, as for the right foot. The image of the metatarsal area, being a reference to the correct longitudinal cavity of the foot. It was different for both feet when measured at rest compared to after exercise. For the heel area, the mean differences in the values between the measurements for the right and left foot was also noted. Additionally, in the author’s study, differences in the pressure on the ground of individual parts of the foot between the right and left foot were noted. Systematic football training, as well as external load and physical effort, affects changes in body balance and the distribution of pressure forces on the sole of the foot. Further studies confirming the results obtained by the authors of this study are the analyses of Bibro M. et al. [29]. They took up the problem of the analysis of the arching and pressure distribution of the plantar side of the feet of young men under the influence of strength training of the lower limbs. The surveyed men were divided into two groups of 30. Group I, subjected to training, completed training in the gym including lower limb exercises within 60 minutes, and group II spent the same period of time passively, in a sitting position. In the group subjected to strength training, in the measurements before and after exercise, the lateral and medial side of the hindfoot were symmetrically loaded, while the load on the forefoot increased significantly, especially on the medial part. One hour of effort also had a slight effect on the height of the arches of both feet. Bogut I. et al. [30] conducted research on the occurrence of foot deformities in city children, as well as on possible generation and sex differences. The results of the research showed that the highest percentage of children did not have a noticeable foot deformity, so more than three-quarters of children in 2005 and 2011 had healthy feet. The only noticeable percentage of children with foot deformities relates to the first-degree flatfoot category, from 9.39% in 2005 and 14.69% in 2011. There were no significant differences in the occurrence of foot deformities between the 2005 and 2011 generations or by gender and age between and within each subgroup. The results of these studies indicate that the largest number of children aged 7–11 years did not have noticeable foot deformities, so in children studied in 2005 and 2011, so most of the children did not have deformities. The only noticeable percentage of children with foot deformity relates to the first-degree flat foot category; however, their percentage was in the range of 9–15%. The boys studied for the purposes of this study were also city dwellers. The results obtained by the authors were not compatible with the studies cited above. The author’s study noted that the total length of the longitudinal arch of both feet of the examined boys showed a tendency to flatten in direct proportion to the age of the examined boys. The arches of the foot differ, however, between the right and left roof. Zdunek M.K. et al. [17] confirmed that people practicing the above disciplines have hollow long arches of the foot. Additionally, the results of the research carried out by the authors showed differences in the distribution of forces on the sole of the foot depending on the sports discipline practiced. In the throwing group, in the right and left feet, the front part of the foot was loaded more frequently, while in the jumping group, the back of the foot was more loaded in the right and left feet. The authors concluded that the observed differences probably resulted from the fact that, due to the specificity of the sports discipline, players have different morphological profiles. The authors of this study also noticed that the studied footballers were mostly characterized by a hollow longitudinal arch. Due to the specifics of their discipline, players are more likely to put stress on the rear of the left foot and the front of the right foot.

## 5. Conclusions

- The total length of the longitudinal arch of both feet of the examined boys showed a tendency to flatten in direct proportion to the age of the examined boys. The arches of the foot differ, however, between the right and left roof. If this tendency is maintained in the left foot, it does not take such a strong direction in the right foot.
- The youngest group of the boys under study showed the greatest deviations of the balance, while the group subjected to training for the longest time (the group of the oldest boys) had distinct smaller deviations of the pressure center.
- In the youngest group of boys, correlations between body balance deviations and waist–hip index were observed.
- The given mean values of the body composition parameters reflect changes with the ontogenetic development, basic somatic parameters (body height and weight) and training experience, and thus with the intensity and volume of training.

Some aspects require further research. The dominant side of the respondents should be taken into account, which may be the reason for the observed differences. The observed correlations may suggest a relationship between body composition parameters and the ability to maintain balance and stabilization performance. 

### Practical Implication

The training rigor supports the proper development of children and has a positive effect on balance and body composition. An important aspect of the training process is not to overtrain the players so that the training is beneficial and supports the natural ontogenetic development.

## Figures and Tables

**Table 1:** Characteristics of the distribution of pressure forces of the foot in the age groups of the studied boys.

	Forefoot LF (%)	Metatarsus LF (%)	Heel LF (%)	Forefoot RF (%)	Metatarsus RF (%)	Heel RF (%)
Footballers aged 8–10 (*n* = 28)						
Me	38.40	0.10	56.70	57.30	0.80	37.95
Q_1_	27.45	0.00	44.70	53.38	0.00	27.15
Q_3_	49.93	2.70	71.80	65.23	6.65	43.65
Footballers aged 11–13 (*n* = 29)						
Me	47.80	0.40	44.80	58.40	9.80	29.20
Q_1_	36.50	0.00	38.10	44.50	2.30	17.60
Q_3_	55.20	11.20	5510	69.40	20.50	49.50
Footballers aged 14–16 (*n* = 33)						
Me	43.70	12.90	40.00	45.30	2.30	49.30
Q_1_	39.00	7.00	37.30	37.30	0.50	43.90
Q_3_	49.20	21.30	45.50	49.70	11.10	56.60

LF—left foot, RF—right foot, Me—median, Q—quartile.

**Table 2:** Characteristics of the changes in the position of the center of gravity examination in the age groups.

	Body COP (mm^2^)	COP LF (mm^2^)	COP RF (mm^2^)
Footballers aged 8–10 (*n* = 28)			
Me	360.62	40.96	73.62
Q_1_	195.05	20.25	25.38
Q_3_	792.63	109.44	245.89
Footballers aged 11–13 (*n* = 29)			
Me	197.84	20.36	33.65
Q_1_	60.96	12.25	12.54
Q_3_	512.23	49.52	123.17
Footballers aged 14–16 (*n* = 33)			
Me	37.76	4.33	7.30
Q_1_	20.71	2.20	2.84
Q_3_	72.57	9.05	14.75

COP—center of pressure, LF—left foot, RF—right foot, Me—median, Q—quartile.

**Table 3:** Significance of differences in parameters of the distribution of pressure forces in the left foot between the studied age groups.

Variable		Forefoot LF			Metatarsus LF			Heel LF		
Kruskal–Wallis test (*p*)		0.100			0.000			0.001		
Age		8–10	11–13	14–16	8–10	11–13	14–16	8–10	11–13	14–16
Test post hoc with the amendment Bonferroni	8–10	X	0.100	0.546	X	1.000	0.000	X	0.122	0.001
	11–13	0.100	X	1.000	1.000	X	0.000	0.122	X	0.321
	14–16	0.546	1.000	X	0.000	0.000	X	0.001	0.321	X

LF—left foot, RF—right foot.

**Table 4:** Significance of differences in parameters of the distribution of pressure forces in the right foot between the studied age groups.

Variable		Forefoot RF			Metatarsus RF			Heel RF		
Kruskal–Wallis test (*p*)		0.000			0.032			0.000		
Age		8–10	11–13	14–16	8–10	11–13	14–16	8–10	11–13	14–16
Test post hoc with the amendment Bonferroni	8–10	X	0.869	0.000	X	0.029	0.776	X	1.000	0.000
	11–13	0.869	X	0.003	0.029	X	0.361	1.000	X	0.000
	14–16	0.000	0.003	X	0.776	0.361	X	0.000	0.000	X

LF—left foot, RF—right foot.

**Table 5:** Significance of the changes in the position of the center of gravity between the studied age groups.

Variable		Body COP			LF COP			RF COP		
Kruskal–Wallis test (*p*)		0.000			0.000			0.000		
Age		8–10	11–13	14–16	8–10	11–13	14–16	8–10	11–13	14–16
Test post hoc with the amendment Bonferroni	8–10	X	0.013	0.000	X	0.155	0.000	X	0.012	0.000
	11–13	0.013	X	0.001	0.155	X	0.000	0.012	X	0.016
	14–16	0.000	0.001	X	0.000	0.000	X	0.000	0.000	X

COP—center of pressure, LF—left foot, RF—right foot.

**Table 6:** Characteristics of selected body composition parameters (InBody) and the level of statistical significance of differences between the investigated footballers classified in age groups.

	TBW	Proteins	Minerals	BFM	FFM	SMM	BMI	PBF	WHR	VFL	Obesity Degree
Footballers aged 8–10 (*n* = 28)											
Me	18.10	4.85	1.80	7.15	24.75	12.65	17.50	22.15	0.77	3.00	106.5
Q1	16.15	4.20	1.47	5.00	22.00	10.98	15.70	18.20	0.75	2.00	99.75
Q3	21.55	5.70	2.06	10.33	29.50	15.43	19.35	27.30	0.79	3.00	111.0
Footballers aged 11–13 (*n* = 29)											
Me	28.20	7.50	2.74	7.60	38.40	20.80	18.30	16.50	0.77	3.00	98.00
Q1	25.90	6.90	2.52	6.10	35.40	18.80	17.10	13.00	0.76	2.00	94.00
Q3	30.80	8.20	2.92	10.50	42.00	22.70	20.10	21.50	0.79	4.00	105.00
Footballers aged 14–16 (*n* = 33)											
Me	37.50	10.10	3.62	6.80	51.30	28.40	20.20	11.30	0.77	2.00	97.00
Q1	33.10	9.10	3.12	5.80	45.20	25.10	18.70	10.20	0.76	2.00	92.00
Q3	43.40	11.70	4.11	9.00	59.20	33.40	21.70	14.90	0.79	3.00	102.00
p	0.000	0.000	0.000	0.757	0.000	0.000	0.001	0.000	0.799	0.411	

TBW—total body water, BFM—total body fat mass, FFM—lean body mass, SMM—skeletal muscle mass, BMI—body mass index, PBF—percentage of adipose tissue, WHR—waist–hip index, VFL—visceral fat.

## Data Availability

The data presented in this study are available on request from the corresponding author.
